# The effects of the glycaemic control on the severity of the delirium in the advanced phase of Alzheimer’s disease

**DOI:** 10.12688/f1000research.26022.1

**Published:** 2020-12-16

**Authors:** Antonio Martocchia, Marta Scarienzi, Pietro Prunas, Enrico Bentivegna, Mauro Cacciafesta, Paolo Martelletti, Giorgio Sesti

**Affiliations:** 1 Sapienza University of Rome, ROMA CAPITALE, RM, 00100, Italy; 2 Department of Cardiovascular, Respiratory, Nephrological, Anesthesiological and Geriatric Sciences, Sapienza University of Rome, Rome, Italy

**Keywords:** Marigliano-Cacciafesta-polypathology-scale, glycaemic control, Alzheimer’s disease, delirium

## Abstract

Background: Behavioural and psychological symptoms of dementia (BPSD) and delirium are common in advanced phases of Alzheimer’s disease (AD).

Methods: Thirty-eight moderate-severe AD patients were enrolled (n=16 affected by type 2 diabetes). Each patient received a comprehensive geriatric assessment (CGA) (including evaluation of BPSD and frailty), and a complete metabolic evaluation (including the measurement of the glycated haemoglobin, HbA1c).

Results: Both the hyper- and hypo-glycaemic extremes of the glycaemic spectrum worsened BPSD, but delirium was more susceptible to hypoglycaemic events. The severity of delirium was significantly related to cognitive function (r = -0.585, p<0.001) and frailty (r = +0.440, p<0.05).

Conclusions: The measurement of HbA1c was useful for evaluating the risk of delirium in relationship to glycaemic control and nutritional status.

## Introduction

Behavioural and psychological symptoms of dementia (BPSD) and delirium are common in the advanced phases of Alzheimer’s disease (AD), whereas cognitive decline is prevalent in the first phases of the disease.

Both the hyper- and hypo-glycaemic extremes of the glycaemic spectrum may worsen cognitive functions in AD patients ^1– 3^, and nutrition plays an important role in the development of the delirium in frail older patients ^4^.

The aim of this preliminary report was to evaluate the relationship between delirium and glycaemic control in the advanced phases of AD.

## Methods

### Ethical statement

The study involved human participants with metabolic syndrome in aging and it was performed in accordance with the ethical standards of the local institutional committee (S. Andrea Hospital, ID number 67910) and with the 1964 Helsinki declaration and its later amendments or comparable ethical standards. Written informed consent for participation and evaluation of data was obtained from the individual participants included in this study or from their caregivers, where ability to consent was affected by delirium.

### Participants

We randomly (systematic sampling) enrolled 38 elderly patients (n = 11 males and n = 27 females) from the outpatients of the S. Andrea Hospital of Rome, in 2019. From their medical records, the patients met the criteria for probable AD dementia proposed by the National Institute on Aging-Alzheimer’s Association (NIA-AA) ^5^ (Mini Mental State Examination (MMSE) score of 12.4+7.3, mean + standard deviation, in the moderate-severe stage of the disease). Prior to inclusion, subjects with other diseases, such as territorial infarction, intracranial haemorrhage, brain tumour, hydrocephalus, or severe white matter hyperintensities (WMH) were excluded from the study. Sixteen of the patients (16/38, 42% of the sample) were affected by type 2 diabetes (T2DM, according to 2020 American Diabetes Association, ADA, criteria) ^6^, undergoing treatment with oral glucose lowering agents (n = 13) or with insulin (n = 3).

### Assessments

Each patient received from our group: a) a comprehensive geriatric assessment (CGA), including the evaluation of the Activity of Daily Living (ADL), the Instrumental Activities of Daily Living (IADL), the Cumulative Illness Rating Scale (CIRS) and the Marigliano-Cacciafesta Polypathology Scale (MCPS) ^7, 8^; b) an anthropometric evaluation, including height, weight, body mass index (BMI), waist circumference, Mini Nutritional Assessment (MNA) ^9^; c) a behavioural and psychological examination, using the Cornell’s scale, the Clinical Dementia Rating scale (CDR), the Neuropsychiatric Inventory (NPI) and the Confusion Assessment Method (CAM, with CAM-severity) ^10– 12^. An assessment of glucose metabolism, including fasting glycaemia and insulinaemia (excluded in insulin-treated patients), glycated haemoglobin (HbA1c) and insulin resistance by the means of the HOMA-index (glycemia mg/dl x insulinaemia µU/mol /405) (excluded in insulin-treated patients) and an evaluation of renal function, measuring creatinine and glomerular filtration rate (GFR) estimated using the Chronic Kidney Disease Epidemiology Collaboration formula (CKD-EPI), was performed using a clinical chemistry analyzer (c16000 Architect System, Abbott Laboratories). A continuous glucose monitoring (FreeStyle Libre system) was used in selected patients with a high risk of hypoglycaemic events, or with great variability of glycaemia during the day.

### Statistical analysis

For the statistical analysis, carried out using Primer of Biostatistics Version 7, one-way analysis of variance (ANOVA) was used for the evaluation of the differences between the groups of patients. The relationship between two variables was examined by the means of regression analysis. A p<0.05 was assumed as significant. Data are presented as mean + standard deviation.

## Results

The clinical characteristics of the 38 AD patients with and without T2DM are described in Table 1 ^13^.

**Table 1.** Clinical characteristics in Alzheimer’s disease (AD) patients with (n = 16, females n = 9) and without (n = 22, females n = 18) type 2 diabetes (T2DM) (mean+standard deviation).

	AD patients without T2DM	AD patients with T2DM
Age (years)	83.3+5.7	82.3+4.2
MMSE	11.6+7.1	12.7+7.5
ADL (IADL M and F)	2.9+2.2 (M: 1.7+1.7; F: 1.2+1.8)	2.7+1.8 (M: 1.4+1.5; F: 0.7+1.0)
MCPS	48.6+10.5	47.4+7.0
CIRS-SI and -CI	2.2+0.6 and 4.8+2.2	2.3+0.4 and 5.6+1.4
BMI	24.4+7.0	26.7+5.7
Waist circumference (cm)	M: 98.7+1.5; F: 90.3+22.6	M: 97.7+8.6; F: 89.1+15.2
MNA	17.4+6.0	18.8+4.8
Cornell	14.2+8.6	11.4+6.0
CDR	2.7+1.4	2.2+1.1
CAM	2.7+1.5	2.2+1.9
CAM-S	7.8+4.1	4.7+3.7 ^*^
NPI	53.7+28.5	40.2+20.2
Fasting glycaemia (mg/dl)	94.5+11.2	135.6+58.0 ^**^
Fasting insulinaemia (µu/ml)	6.0+2.6	8.9+9.5
HbA1c	5.4+0.4%	6.8+1.1% ^***^
HOMA index	1.4+0.6	3.2+3.4 ^*^
GFR (CKD-EPI; ml/min)	66.0+16.9	64.9+23.4

MMSE, Mini Mental State Examination; ADL, Activity of Daily Living; IADL, Instrumental Activities of Daily Living; MCPS, Marigliano-Cacciafesta Polypathology Scale; CIRS, Cumulative Illness Rating Scale; SI, severity index; BMI, body mass index, MNA, Mini Nutritional Assessment; CDR, Clinical Dementia Rating scale; CAM, Confusion Assessment Method; CAM-S, CAM-severity; NPI, Neuropsychiatric Inventory; HbA1c, glycated haemoglobin; GFR, glomerular filtration rate; CKD-EPI, Chronic Kidney Disease Epidemiology Collaboration formula; M, male; F, female. *p<0.05, **p<0.01, ***p<0.0001.

**Figure 1.**
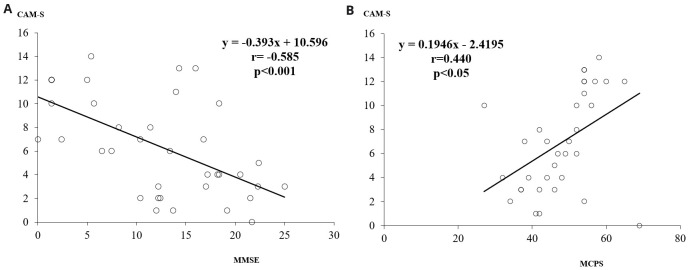
**a**) The relationship between MMSE and CAM-S scores. **b**) The relationship between MCPS and CAM-S scores. MMSE, Mini Mental State Examination; CAM-S, Confusion Assessment Method-severity; MCPS, Marigliano-Cacciafesta Polypathology Scale.

The MMSE scores were inversely and significantly related to CAM-S score (r = -0.585, p<0.001), while MCPS scores were positively related to the CAM-S score (r = 0.440, p<0.05) ( Figure 1a and 1b).

**Figure 2.**
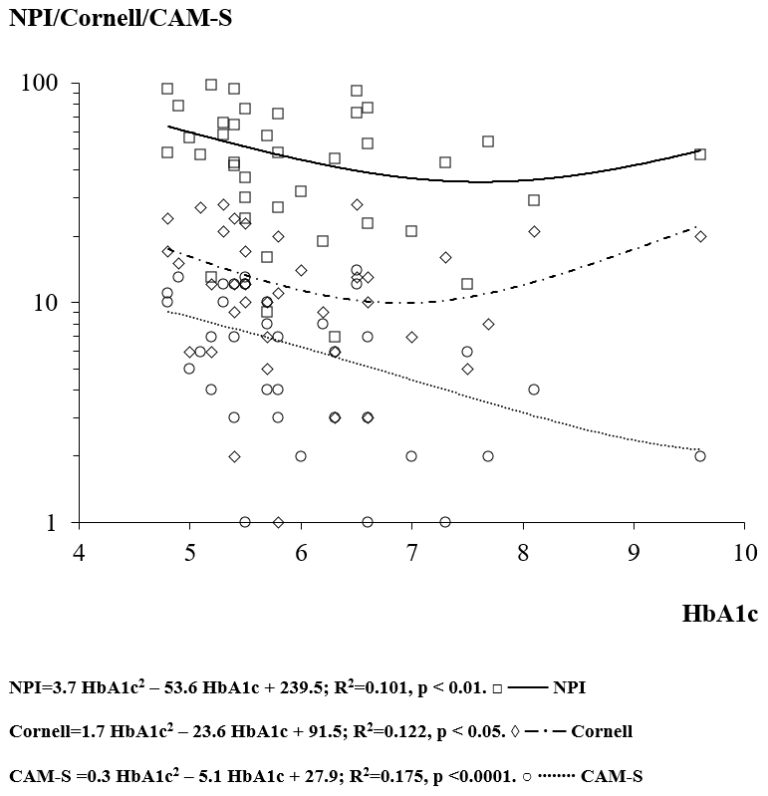
The relationship between the glycated haemoglobin (HbA1c) and the Neuropsychiatric Inventory (NPI), Cornell’s scale and Confusion Assessment Method-severity (CAM-S) score.

The regression analysis between HbA1c and MMSE score showed a concave downward “ **∩**” parabola (not significant) with apex at HbA1c of 10.5% and MMSE of 13.0. The regression analysis between HbA1c and the psychometric BPSD scales (Cornell, NPI and CAM-S) showed a “U” shape (concave upward parabola) (Cornell p<0.05, NPI<0.01 and CAM-S p<0.0001) ( Figure 2). The apices of the parabolas were at HbA1c of 6.9%, 7.6% and 10.3%, the levels that minimized the Cornell, NPI and CAM-S scores, respectively ( Figure 2). Therefore, the mean HbA1c that minimized all the behavioural disorders was 8.3%.

**Figure 3.**
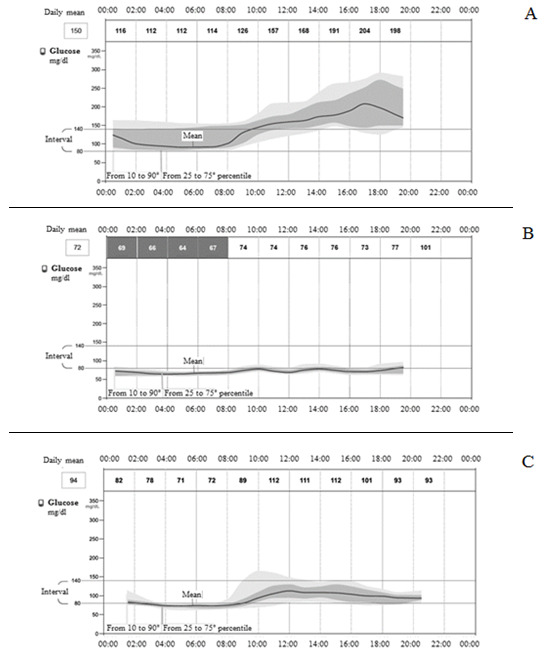
Continuous glucose monitoring in selected cases (n=3). **A**) Patient A, 81 years, MMSE=1.5, NPI=77, Cornell=13, CAM-S=17, HbA1c=6.6%, glucose mean 150 mg/dl, above/in/under the interval 56/43/1%, event of low glucose=0, mean duration=0. **B**) Patient B, 77 years, MMSE=13.7, NPI=30, Cornell=12, CAM-S=4, HbA1c=5.5%, glucose mean 72 mg/dl, above/in/under the interval 0/16/84%, event of low glucose=26, mean duration=296 min. **C**) Patient C, 82 years, MMSE=10.5, NPI=52, Cornell=18, CAM-S=11, HbA1c=5.7%, glucose mean 94 mg/dl, above/in/under the interval 7/54/39%, event of low glucose=4, mean duration=281 min.

Continuous glucose monitoring in selected patients (n = 3) showed that, even if HbA1c level was within the normal range, either a great variability in glycaemia during the 24 hours or repeated hypo-glycaemic events during the night (up to 26 hypoglycaemic events in a single patient) were present ( Figure 3).

## Discussion

In our preliminary study, polypathology and frailty was associated with the severity of delirium as demonstrated by using the MCPS scale in correlation with the CAM-S scale.

To the best of our knowledge this is the first report about the association of the MCPS and CAM-S scale, whereas the MCPS was previously evaluated by our group in patients with comorbidities and frailty ^8^.

Glycaemic control was associated with modification of BPSD and delirium in the advanced phases of AD. In particular, delirium (as indicated by the CAM-S score) was more susceptible to the glycaemic control in its low range. As considered by point 7 (Metabolism and Nutritional state) of MCPS, high glucose levels increase the score of the MCPS scale (+25) only in conditions of diabetes mellitus decompensation, with significant risks of development of delirium.

Glycaemic control modulated the appearance of BPSD and delirium with a polynomial U-shaped curve, with both the hyper- and hypo-glycaemic extremes of the glycaemic spectrum worsening BPSD. The regression equations showed that HbA1c levels between 6.9–10.3% (mean 8.3%) minimized BPSD, in agreement with International Diabetes Federation (2020) ^14^ and ADA (2020) ^6^ guidelines for managing T2DM in older people. As matter of fact, in healthy older subjects (with few coexisting chronic illnesses, intact cognitive and functional status) a reasonable HbA1c goal is <7.5% (with fasting or preprandial glucose of 90–130 mg/dl), whereas in complex/poor health patients (with more chronic illnesses, moderate-to severe cognitive impairment or >2 ADL dependencies) the reasonable HbA1c goal rises to <8.5 (with fasting or preprandial glucose of 100–180 mg/dl) to avoid possible overtreatment, hypoglycaemic events and fall risk.

The HbA1c value of 6% corresponds to a mean glucose of 126 mg/dl, with 95% prediction limits of 100–152 mg/dl, which includes the lower goals for glycaemia (100–180 mg/dl) suggested by ADA standard of care ^15– 17^ in older frail patients with moderate-severe dementia. More than half of our patients (22/38, 58%) showed HbA1c levels lower than 6%.

The reduction of one point (%) of HbA1c (from 6.8% to 5.8%) was associated with an increase of +2.2 points in Cornell, +7.0 points in NPI and +0.2 in CAM-S scores. Because of the parabolic profiles of the curves, another reduction of one point of HbA1c (from 5.8% to 4.8%) resulted in an exponential increase of BPSD scores (+5.6 Cornell, +14.4 NPI and +2.6 CAM-S). 

The variability of glycaemia seemed to be associated with greater BPSD in AD patients, although it was not possible to carry out a statistical analysis because of the few number of patients in the sample.

In conclusion, the measurement of HbA1c in elderly AD patients with advanced dementia (with and without T2DM) is recommended in order to evaluate glycaemic control (and nutritional status). The reduction of mean glycaemic levels (due to malnutrition in non-diabetic patients or overtreatment in diabetic patients) should be avoided by means of a multidimensional approach.

## Data availability

DANS EASY: The Glycaemic Control and the Severity of the Delirium. https://doi.org/10.17026/dans-xq4-58fq ^13^.

Data are available under the terms of the Creative Commons Zero "No rights reserved" data waiver (CC0 1.0 Public domain dedication).
