# Supplementary material for: The effects of the glycaemic control on the severity of the delirium in the advanced phase of Alzheimer’s disease
Source: F1000Res. 2021 Jul 28;9:1470. Originally published 2020 Dec 16. [Version 2] doi: 10.12688/f1000research.26022.2 (PMC8278245; doi:10.12688/f1000research.26022.2)
Supplement: Peer review history

# Peer review: Reviewer response for version 1

**DOI:** 10.5256/f1000research.28717.r89381

**Published:** 2021-07-13

**Authors:** Takahiko Nagamine

**Affiliations:** 1 Director of Sunlight Brain Research Center, Hofu, Japan; 2 Department of Emergency Medicine, Matsumoto Surgical Hospital, Hofu, Japan

This paper analyzes HbA1c (blood glucose control status) and delirium in patients with Alzheimer's disease. It is interesting to note that both high and low blood glucose levels increase the risk of delirium. The paper is clinically significant because it shows that HbA1c as a predictor of delirium may be valid. 

 If possible, two points should be considered: one is to conduct a study with separate analysis for the presence or absence of diabetes mellitus, and the other is to design the study to take into account the effects of antipsychotics and sleeping pills on blood glucose and delirium.

 Alzheimer's disease is caused by a decrease in neurotransmission in the acetylcholine system. The central acetylcholine nervous system may be less responsive to glucose loading. Older patients with Alzheimer's disease may occasionally experience hypoglycemia. This is due not only to the decline in acetylcholine and other neurotransmitters in Alzheimer's disease itself but also to antipsychotic drugs used for delirium ^1^.

 The level of HbA1c is also affected by the type of hypoglycemic drug. For example, concomitant use of insulin secretagogues (e.g., sulfonylureas) and dopamine blockers can cause hypoglycemia and delirium-like disturbances in consciousness ^2^.

 To avoid the effect of diabetic drugs, it is recommended to divide Alzheimer's disease into two groups, one with comorbid diabetes and the other without diabetes, and study the relationship between HbA1c and delirium in each group. Delirium is influenced by Alzheimer's disease, but also by medications used to treat insomnia (e.g., benzodiazepine receptor agonists), so it is advisable to adjust for medications that have a risk of inducing delirium as a background factor ^3^. 

 Even though there are confounding factors, the study of HbA1c as a predictor of delirium is very unique and a good perspective from the point of view that Alzheimer's disease is called diabetes of the brain.

Is the work clearly and accurately presented and does it cite the current literature?

Yes

If applicable, is the statistical analysis and its interpretation appropriate?

Partly

Are all the source data underlying the results available to ensure full reproducibility?

Yes

Is the study design appropriate and is the work technically sound?

Yes

Are the conclusions drawn adequately supported by the results?

Yes

Are sufficient details of methods and analysis provided to allow replication by others?

Yes

Reviewer Expertise:

psychopharmacology, emergency medicine, diabetes

I confirm that I have read this submission and believe that I have an appropriate level of expertise to confirm that it is of an acceptable scientific standard.

## References

[1] : Severe hypoglycemia in an elderly patient withAlzheimer's disease and with sepsis: The role of neurotransmitters on glucose regulation. *Geriatrics & Gerontology International* .2021;21(1) : 10.1111/ggi.14091 116-117 10.1111/ggi.14091 33200528

[2] : Severe Hypoglycemia Associated with Tiapride in an Elderly Patient with Diabetes and Psychosis. *Innov Clin Neurosci* .14(1-2) :12-13 28409061PMC5373790

[3] : Long‐term preoperative use of zolpidem is associated with postoperative delirium in elderly patients undergoing hip surgery. *Psychogeriatrics* .2021;21(4) : 10.1111/psyg.12690 689-690 10.1111/psyg.12690 33848031

# Response

**Published:** 2021-07-22

**Authors:** Antonio Martocchia

**Affiliations:** S.Andrea Hospital Sapienza University of Rome, Italy

Dear Prof Takahiko Nagamine,

 We agree with the evidence about HbA1c as a predictor of delirium in Alzheimer’s disease and with the interest about the effects of the presence/absence of diabetes mellitus or concurrent therapies (antipsychotics and sleeping pills, as well as insulin secretagogues, e.g. sulfonylureas, and dopamine blockers) on HbA1c and delirium.

 In our sample of AD patients with diabetes, one subject was treated with repaglinide (we added a sentence in the text in methods).

 The percentage of use of drugs (antipsychotics, sleeping pills, antidepressants, antidementia, antiparkinsonian agents, mood stabilizing medications) in AD patients with or without diabetes, were respectively: 37%, 19%, 19%, 6%, 19%, 6%, and 32%, 18%, 23%, 9%, 9%, 9% (differences not significant) (we added a sentence in the text, results and discussion). One AD patients with diabetes was treated with zolpidem. We added the suggested references in the bibliography’s list.

 Yours sincerely, Antonio Martocchia, MD PhD

# Peer review: Reviewer response for version 1

**DOI:** 10.5256/f1000research.28717.r85330

**Published:** 2021-05-17

**Authors:** Sandra Sigala

**Affiliations:** 1 Section of Pharmacology, Department of Molecular and Translational Medicine, University of Brescia, Brescia, Italy

The Ms entitled "The effects of the glycaemic control on the severity of the delirium in the advanced phase of Alzheimer’s disease" by Martocchia and coll is very interesting and gives support to the evidence that glycemic control in the frail elderly is important in slowing cognitive degeneration/Alzheimer's onset.

 Authors highlighted as well the importance in these patients of a comprehensive clinical evaluation, which includes both biochemical and functional values.

 The main limit of the study is the sample size, but this point has been taken up and discussed by the Authors themselves.

 I have only minor points to underline:

1. The first sentence in the Introduction needs a reference
2. Characters in the Figure legend need to be unified - Figure 3 is hard to read
3. Throughout the Ms, please correct typos and the language style

Is the work clearly and accurately presented and does it cite the current literature?

Yes

If applicable, is the statistical analysis and its interpretation appropriate?

Yes

Are all the source data underlying the results available to ensure full reproducibility?

No source data required

Is the study design appropriate and is the work technically sound?

Yes

Are the conclusions drawn adequately supported by the results?

Yes

Are sufficient details of methods and analysis provided to allow replication by others?

Yes

Reviewer Expertise:

Clinical pharmacology; translational oncology

I confirm that I have read this submission and believe that I have an appropriate level of expertise to confirm that it is of an acceptable scientific standard.

# Response

**Published:** 2021-07-22

**Authors:** Antonio Martocchia

**Affiliations:** S.Andrea Hospital Sapienza University of Rome, Italy

Dear Reviewer Prof Sandra Sigala,

 We really appreciated your review, discussing the importance of a comprehensive clinical evaluation in frail elderly with Alzheimer’s disease.

 We agree that the limit of study is the sample size: we completed the sentence in the manuscript in the Discussion: “The variability of glycaemia seemed to be associated with greater BPSD in AD patients, although it was not possible to carry out a statistical analysis because of the few number of patients in the sample (a limit of the study)”.

 We added the reference (1) and modified the first sentence: “Behavioral and psychological symptoms of dementia (BPSD) and delirium are common in the advanced phases of Alzheimer’s disease (AD) and worsen with disease severity, whereas symptoms of cognitive decline may be prevalent in the first phases of the disease (1)”.

 We unified the characters in the figure legends.

 We modified the figure 3.

 We corrected typos and the language style throughout the Ms.

 Yours sincerely Antonio Martocchia, MD PhD
